# POST-REPERFUSION LIVER BIOPSY AND ITS VALUE IN PREDICTING MORTALITY AND GRAFT DYSFUNCTION AFTER LIVER TRANSPLANTATION

**DOI:** 10.1590/0102-6720201600030014

**Published:** 2016

**Authors:** Marcos Vinícius ZANCHET, Larissa Luvison Gomes da SILVA, Jorge Eduardo Fouto MATIAS, Júlio Cezar Uili COELHO

**Affiliations:** Postgraduate Program im Sugical clinics, Healtu Sciences Sector, Federal University of Paraná, Curitiba, PR, Brazil.

**Keywords:** Hepatic Biopsy, Reperfusion, Liver Transplant, .Primary Graft Dysfunction, Complications, Survival

## Abstract

**Background:**

The outcome of the patients after liver transplant is complex and to characterize the risk for complications is not always easy. In this context, the hepatic post-reperfusion biopsy is capable of portraying alterations of prognostic importance.

**Aim:**

To compare the results of liver transplantation, correlating the different histologic features of the hepatic post-reperfusion biopsy with graft dysfunction, primary non-function and patient survival in the first year after transplantation.

**Method:**

From the 377 transplants performed from 1996 to 2008, 164 patients were selected. Medical records were reviewed and the following clinical outcomes were registered: mortality in 1, 3, 6 and 12 months, graft dysfunction in varied degrees and primary graft non-function. The post-reperfusion biopsies had been examined by a blinded pathologist for the outcomes. The following histological variables had been evaluated: ischemic alterations, congestion, steatosis, neutrophilic exudate, monomorphonuclear infiltrate and necrosis.

**Results:**

The variables associated with increased mortality were: steatosis (p=0.02209), monomorphonuclear infiltrate (p=0.03935) and necrosis (p<0.00001). The neutrophilic exudate reduced mortality in this study (p=0.00659). The primary non-function showed significant association (p<0.05) with the necrosis, steatosis and the monomorphonuclear infiltrate.

**Conclusion:**

Post-reperfusion biopsy is useful tool to foresee complications after liver transplant.

## RESUMO

**Racional:**

A evolução dos pacientes após transplante hepático é complexa e caracterizar o risco para complicações nem sempre é fácil. Nesse contexto, a biópsia hepática pós-reperfusão é capaz de retratar alterações de importância prognóstica.

**Objetivo:**

Avaliar os resultados no primeiro ano após transplante hepático, correlacionando as alterações histológicas à biópsia hepática pós-reperfusão com a sobrevida, a disfunção e o não-funcionamento primário do enxerto.

**Método:**

Dos 377 transplantes ocorridos de 1996 a 2008, 164 pacientes foram selecionados para estudo. Os seguintes desfechos clínicos foram registrados: mortalidade em 1, 3, 6 e 12 meses, disfunção do enxerto em graus variados e o não-funcionamento primário do enxerto. As biópsias pós-reperfusão foram examinadas por um patologista sem conhecimento dos resultados. As seguintes variáveis histológicas foram avaliadas: alterações isquêmicas, congestão, esteatose, exsudato neutrofílico, infiltrado monomorfonuclear e necrose.

**Resultados:**

As variáveis associadas com aumento da mortalidade foram: esteatose (p=0.02209), infiltrado monomorfonuclear (p=0.03935) e necrose (p<0.00001). O infiltrado neutrofílico reduziu a mortalidade neste estudo (p=0.00659). O não-funcionamento primário do enxerto mostrou associação significativa (p<0.05) com a necrose, a esteatose e com o infiltrado monomorfonuclear.

**Conclusão:**

A biópsia hepática pós-reperfusão é ferramenta útil em prever complicações após o transplante hepático.

## INTRODUCTION

Liver transplantation (LT) is accepted as an effective modality of treatment for patients with end-stage liver disease. The evolution after LT is complex, given the many complications that may occur early or late. In this context, the post-reperfusion liver biopsy (PRB) allows to evaluate changes present not only in the donor but also those arising in organ preservation period and after its implant.

The aim of this study was to assess the impact of histological alterations in PRB in the first year after LT, comparing their relationship to survival and graft dysfunction.

## METHOD

This study is based on retrospective analysis of patients undergoing orthotopic cadaveric liver transplantation selected among all transplants performed in 2008 from 1996 in Liver Transplant Service, Hospital de Clínicas, Federal University of Paraná, Curitiba, PR, Brazil. It was approved by the Human Research Ethics Committee of the institution under registration CAAE 0036.0.208.000-09, CEP 1871.038 / 2009-2.

The following data were recorded: age; gender; cause of cirrhosis; ischemia time of the graft; mortality at 1, 3, 6, 12 months after transplantation; graft dysfunction (mild and severe) and primary graft non-function (PNF).

The exclusion criteria were: living-donor transplants, double-type (liver and kidney), "domino", with hepatic reduction, emergency transplantation, retransplantation, pediatric patients and those whose records or biopsy could not be assessed.

All biopsies were performed by a wedge-shaped incision in the liver edge after the graft implant and its reperfusion and before abdominal closure. They were stored in 10% formalin, embedded in paraffin and stained with hematoxylin and eosin. They had been prospectively examined by a blinded pathologist for the outcomes. The features studied were: necrosis, macrovesicular steatosis, monomorphonuclear inflammatory infiltrate, neutrophilic exudate, ischemic alterations and congestion, as shown in Table 1.

**TABLE 1:** Histological features of PRB and their graduation

Cellular Necrosis	Absent	Mild	Moderate	Severe
Macrovesicular Steatosis	Absent	Mild or ≤33%	Moderate or 34%-66%	Severe or >66%
Monomorphonuclear Inflammatory Infiltrate	Absent	Mild	Moderate	Severe
Neutrophilic Exudate	Absent	Mild	Moderate	Severe
Ischemic Alterations	Absent	Mild	Moderate	Severe
Congestion	Absent	Present		

PRB=post-reperfusion liver biopsy

The assessment and classification of graft dysfunction was based on laboratory tests and postoperative outcome. Patients with PNF met the UNOS criteria for adults: setting ≤10 days; AST≥5.000 IU/l and one of the following: a) INR≥3.0; b) acidosis (pH≤7.3 or lactate≥2X normal values). Since this is a retrospective evaluation, PNF was associated with death or need for retransplantation. The remaining cases of dysfunction that did not progress to graft failure were classified as mild or severe, according to the following data: 1) mild dysfunction was diagnosed when presented elevated aminotransferases (AST or ALT) above 2.000 IU/l in the first week after LT; and 2) severe dysfunction was diagnosed when presented elevated aminotransferases (AST or ALT) above 2.000 IU/l and increased INR ≥ 2.5 in the first week after LT.

### Statistical analysis

The chi-square test was used for binomial variables and the Kruskal-Wallis test for multinomial variables. The variables were classified in ratio of frequencies and the level of significance of 5% was adopted (p≤0.05). Still, the Spearman coefficient was performed for positive or negative correlation among biopsy variables each other and among the clinical outcomes. For statistical purposes, each parameter was assessed as an independent variable.

## RESULTS

Of the 377 transplants performed in the period, 164 were selected for the study. In all, 213 patients were excluded: 46 pediatric; 43 living donor transplants; 28 emergency transplants (including retransplantation), six double-type transplants; two with liver reduction; two "domino"; and 86 whose records or biopsy could not be assessed.

The average age was 46.52±12.22 years (18-70), 100 men (61%) and 64 women (39%). The main causes included: C-hepatitis (34%); alcohol (21%); B-hepatitis (13%); cholestatic diseases (10%); autoimmune (9%); cryptogenic (5%); others (8%).

The warm ischemia time was, on average, 55.07±17.27 min and cold ischemia time 373.92±151.57 min.

In the first year after LT, mortality of selected patients with evaluable biopsy was 21.95%.

### Ischemic alterations

There were no patients without ischemic alterations on biopsy. Mild alterations were found in 117 patients, moderate in 47 and none had severe ischemic alterations. When comparing these two groups in terms of mortality, no significant differences were observed in the considered periods.

### Congestion

There were 155 patients without and nine with congestion. Mortality was similar between these groups.

### Steatosis

There were 86 patients with no evidence of steatosis in PRB. Mild steatosis was found in 71 and moderate in seven. None had severe steatosis. Mortality related to this variable was significantly higher in the group with moderate steatosis compared to the groups with mild and no steatosis, which showed similar mortality. It occurred only in the first month after the LT (p=0.02209). In other periods, mortality was similar among the groups.

### Neutrophilic exudate

Only one patient had no exudate in biopsy. In all, 127 had mild exudative alterations and 35 moderate alterations. Only one had severe alterations. For analytical purposes, were considered only two groups of patients. The group with no neutrophilic exudate (n=1) was incorporated into the group with mild neutrophilic exudate, and the group with severe neutrophilic exudate (n=1) was incorporated into the group with moderate neutrophilic exudate. After this correction, the mortality of these final two groups - one with 128 and the other with 36 patients - was assessed. It was found an inverse correlation with mortality. In the four periods considered, mortality was higher in patients with mild neutrophilic infiltrate and lower in patients with moderate infiltrate. The higher the degree of neutrophilic exudate, lower the mortality.

### Monomorphonuclear infiltrate 

There were 128 patients without detectable alterations; 33 with mild infiltrate; and three with moderate infiltrate. None showed severe infiltrate. For statistical analysis, patients were rearranged according to the presence or absence of infiltrate in biopsy. Two new groups of 36 and 128 patients, respectively, had been formed. The mortality was significantly higher in patients with infiltrate on biopsy in the first month (p=0.03935) and at six months (p=0.04312) after LT.

### Cellular necrosis 

One hundred and three patients showed no cellular necrosis; 48 had mild necrotic alterations, 13 moderate, and none had severe alterations. Mortality was significantly higher in patients with moderate necrosis in all periods, but was similar among patients with mild and without necrosis (Table 2).

**TABLE 2:**
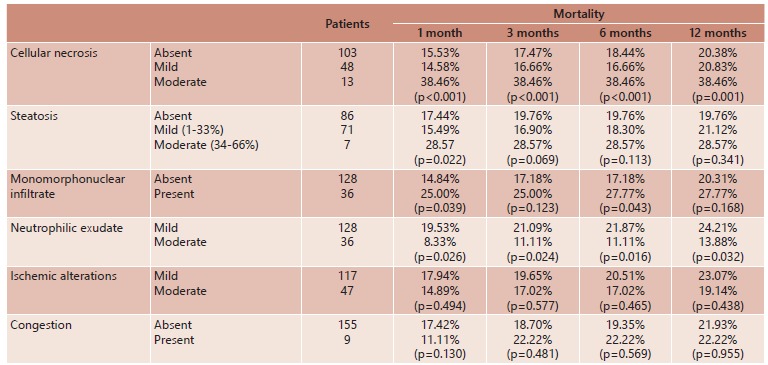
Histological features of PRB and their statistical relationships with mortality in the first year after LT

Analysis performed using the R software, version 2.11.1, Copyright (C) 2010 The R Foundation for Statistical Computing ISBN 3-900051-07-0. PRB=post-reperfusion liver biopsy; LT=liver transplantation.

In the 164 patients studied, PNF occurred in 8.28% (n=13). There were 76 cases of mild graft dysfunction (46.34%) and 40 cases of severe dysfunction (26.21%). In patients without dysfunction, the chance to develop PNF was zero. In patients with mild and severe dysfunction, the chance was 17.1% and 32.5% respectively.

All patients with PNF had both biochemical changes (aminotransferases and INR enlargement). In these, PNF was the next step in the journey of liver dysfunction, progressing from mild to severe and from severe to graft failure. Thus it was considered a multinomial variable, represented in increasing degree of severity, as follows: 1) no dysfunction; 2) mild dysfunction; 3) severe dysfunction; 4) PNF. Table 3 emphasizes the histological features that significantly correlated with graft dysfunction (p <0.05).

**TABLE 3:** Graft dysfunction occurrence according to PRB

Histological features	Statistical significance	
	Kruskal-Wallis	p
Ischemic alterations	0.004	0.94590
Congestion	0.091	0.76205
Steatosis	5.527	0.06306
Neutrophilic exudate	2.769	0.42859
Monomorphonuclear infiltrate	5.204	0.07411
Cellular necrosis	9.369	0.00923

Analysis performed using the R software, version 2.11.1, Copyright (C) 2010 The R Foundation for Statistical Computing ISBN 3-900051-07-0. PRB = post-reperfusion liver biopsy.

As stated, the only variable with significant correlation with dysfunction was liver necrosis. Steatosis - a recognized risk factor for dysfunction - showed an increasing trend, but without statistical significance. The same was found for monomorphonuclear infiltrate. Table 4 shows the effects of necrosis on graft function, with values ​​ranging from 1 (normal) to 4 (failure). 

**TABLE 4:** Occurrence of PNF according to histological features of the PRB

Groups	Patients	PNF
Ischemic alterations - Mild	111	9,01%
Ischemic alterations - Moderate	46	6,52%
p-value		0,42905
Congestion - Absent	149	8,72%
Congestion - Present	8	0
p-value		0,00021
Steatosis - Absent	84	7,14%
Steatosis - Mild	67	7,46%
Steatosis - Moderate	6	33,33%
p-value		<0,00001
Neutrophilic exsudate - Mild	122	9,01%
Neutrophilic exsudate - Moderate	35	5,71%
p-value		0,28102
Monomorphonuclear infiltrate - Absent	122	5,73%
Monomorphonuclear infiltrate - Present	35	17,14%
p-value		0,00281
Necrosis - Absent	99	8,08%
Necrosis - Mild	45	6,66%
Necrosis - Moderate	13	15,38%
p-value		0,00123

Analysis performed using the R software, version 2.11.1, Copyright (C) 2010 The R Foundation for Statistical Computing ISBN 3-900051-07-0. PNF=primary non-function; PRB=post-reperfusion liver biopsy.

However, by considering the PNF as binomial variable (present or absent), the results were different. When the patients were stratified according to ischemic alterations, hepatic congestion or neutrophilic exudate, there were no significant differences in rates of PNF. When stratified according to steatosis, higher rates were found in the group with moderate steatosis, but similar lower rates in the groups with mild and without steatosis. Respect to monomorphonuclear infiltrate, there was an increase in PNF according to the presence of infiltrate. And respecting cellular necrosis, higher PNF rates were observed in the group with moderate necrosis, but lower and similar rates in the groups with mild and without necrosis (Table 4).

## DISCUSSION

The characterization of graft dysfunction and PNF is a complex task. The theoretical definition varies among authors and transplant centers in the world^2^. In our center, PNF is defined as "graft failure that occurs in the first 90 days after transplantation and cannot be clearly associated to technical factors or histological evidence of rejection." The definitions in the literature follow with its own terminology and classification and not always emphasize and distinguish mild and severe forms. Table 5 illustrates some definitions for diagnosis of graft dysfunction.

**TABLE 5:**
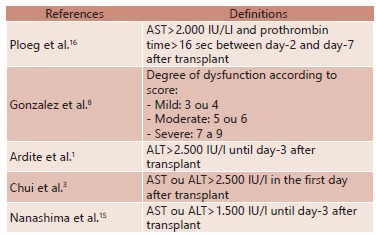
Definitions for diagnosis of graft dysfunction

The classification allows separating two groups of patients. The first, with mild dysfunction of the graft, had a kind of "reperfusion hepatitis", with the aminotransferases elevation as the main alteration. The second, with severe dysfunction, presented hepatic biosynthesis problems, which resulted in decreased production of clotting factors and elevation of INR.

The biopsy analysis is complex and depends on the experience of a dedicated pathologist. The characterization of polymorphonuclear infiltrate, described here as neutrophilic exudate, is sometimes a difficult task. In this study, it was proposed a more panoramic and global assess of the biopsy specimen, not only of the deepest areas, such as found in needle biopsy. Three studies^7^ ^,^ ^12^ ^,^ ^13^ reported a significant association of inflammatory infiltrate with graft dysfunction and poor outcome. Kakizoe et al.^12^ showed no increased risk of dysfunction and mortality according to any of the features studied in fine-needle aspiration biopsy, but observed that patients with severe infiltration and zonal necrosis are subject to poor outcome. Gaffey et al.^7^ reported association of preservation injury with histological inflammation in needle biopsies. Koçbıyık et al.^13^ emphasize that the assessment of neutrophilic infiltrate must cover deeper areas of the liver, beyond the subcapsular space. Also, the presence of subcapsular infiltrate is properly related to mechanical trauma of the liver during the operation, but not with the graft function and survival. Contradicting these studies, it was found that the higher degree of neutrophilic exudate decreases mortality and does not correlate with dysfunction and PNF. However, the deeper areas of the parenchyma were not assessed.

Other features in biopsy that impact on mortality and graft dysfunction were macrovesicular steatosis, monomorphonclear infiltrate and cellular necrosis.

By categorizing the graft dysfunction as multinomial variable, only the hepatic necrosis showed significant correlation.

However, considering the graft failure as a binomial variable (presence or not), the results were different. Three variables showed significant correlation: necrosis, steatosis and monomorphonuclear infiltrate. Mild necrosis did not increase graft failure compared to the absence of necrosis, but there was significant increase in moderate necrosis (15.38% vs. 6.66%, p=0.001). The same was found for steatosis, with increase only in moderate degree (33% vs. 7.46%, p<0.00001). With regard to infiltrate, it almost tripled the incidence of PNF (17.14% vs. 5.73%, p=0.00281).

No significant mortality was associated with mild steatosis (17.4% vs 15.5%). Only moderate steatosis was associated with higher mortality (28.5%), and only in the first month after LT (p=0.02). Several other authors^9^ ^,^ ^18^ ^,^ ^19^ ^,^ ^20^ ^,^ ^24^ support these results. Also, the occurrence of PNF was significantly higher in patients with moderate steatosis compared to mild steatosis (33.33% vs. 7.46%, p<0.00001). It has been proposed that a high degree of steatosis impairs graft function by altering membrane permeability and microcirculation^6^ ^,^ ^18^ ^,^ ^20^. 

The presence of monomorphonuclear infiltration increased mortality. Statistical significance was observed only in the first and sixth months after LT. Also, there was a higher incidence of PNF (17.14% vs. 5.73%, p=0.00281). The highest mortality in the first month after LT is mostly associated to high rates of PNF.

The injury following the ischemia-reperfusion process, known as preservation injury, leads to failure of the hepatic microcirculation, characterized by sinusoidal hemoconcentration, sinusoidal leukocyte stasis, sinusoidal narrowing, reduced perfusion pressure and hypoxic injury of the endothelial cells^17^ ^,^ ^22^. Yu et al.^25^ emphasize that any injury to the liver during its collection, preservation and implant can lead to it, and highlighted as the leading cause of PNF. Interestingly, the authors reported no consistent histological criteria for this alteration.

In vivo analysis of postischemic hepatic microcirculation revealed the induction of leukocyte-endothelial cell interaction within sinusoids and postsinusoidal venules and concomitant sinusoidal perfusion deficits associated with tissue hypoxia^22^. The mechanism appears to be related to increased flow resistance imposed by the accumulation of leukocytes in the microcirculation ^5^ ^,^ ^23^. Ferguson et al.^5^ studied the spatial relationship between the accumulation of white blood cells and microvascular injury during post-ischemic reperfusion. In vivo studies in rats using epifluorescent videomicroscopy demonstrated that the level of leukocyte accumulation in the liver correlated well with microvascular injury.

Other studies have assessed the effects of post-ischemic reperfusion injury and leukocyte infiltration in the liver microcirculation^17^ ^,^ ^22^ ^,^ ^23^. They demonstrate that reperfusion results in increased leukocyte stasis, leading to increased resistance to capillary flow. In the liver submitted to ischemia and reperfusion, about 27% of sinusoids are not able to resume blood flow ("no-reflow") compared to sinusoidal without leukocyte stasis.

It was not aim of the present study to characterize the sinusoidal leukostasis, but only the presence of polymorphonuclear and monomorphonuclear leukocytes in the hepatic parenchyma as a whole. Differently, it was categorized the type of white blood cell in the biopsy specimen and assessed as an independent variable. The presence of monomorphonuclear leukocytes - but not polymorphonuclear - was associated with increased risk of PNF and higher mortality. Regarding the hepatic microcirculation, Puhl et al.^17^ and Vollmar et al.^22^ pointed out that reactive hyperemia is one of the compensatory mechanisms for the perfusion deficit after ischemic injury. This response was observed in animals^21^, in humans^22^ and also in the transplanted liver^17^. It is through the reactive hyperemia that perfusional balance returns in the sinusoids of the transplanted liver, resulting in lower elevation of aminotransferases and bilirubin^17^.

It has been postulated that a prolonged cold ischemia time was associated with more damage to the hepatic sinusoids ^11^ ^,^ ^14^, while the warm ischemia affects primarily the endothelial cells^11^. As soon as the liver is removed from the donor, it suffers ischemia, which induces intracellular and extracellular changes, such as membrane alterations, edema, calcium overload, acidosis, oxidation, cytokines release, apoptosis, and disorders in the microcirculation^4^.

This study did not identify a statistically significant correlation between ischemic alterations and mortality, and also with dysfunction and PNF. It was not aim of the study to evaluate the reactive hyperemia in response to ischemia. It was found that ischemic alterations are a reversible and universal phenomenon, which occurs in several degrees and not always impact in graft function. However, it is noteworthy that no patient presented with severe ischemic alterations on biopsy, which could change the results.

Cellular necrosis - a consecrated factor of poor prognosis - was one of the features that most significantly associated with mortality and graft dysfunction. Patients with moderate cellular necrosis had higher rates of mortality after LT when compared to patients with mild or no necrosis, which showed similar rates. In an animal model^10^, liver viability following ischemia-reperfusion was inversely related to cellular necrosis. Other authors reported similar findings in humans^7^ ^,^ ^12^ ^,^ ^13^.

## CONCLUSIONS

Mortality had a significant correlation with steatosis, monomorphonuclear infiltrate and cellular necrosis, with proportional increase according to histological worsening. However, moderate neutrophilic exsudate reduced mortality. The only feature that correlates significantly with graft dysfunction was cellular necrosis. Thus, the post-reperfusion biopsy is a useful tool to predict complications after liver transplantation.
